# The Effects of Varicocelectomy on the Patients With Premature Ejaculation

**DOI:** 10.5812/numonthly.15991

**Published:** 2014-05-03

**Authors:** Amir Abbas Asadpour, Mohammad Aslezare, Lina Nazari Adkani, Mohsen Armin, Mohammad Vojdani

**Affiliations:** 1 Department of Urology, Faculty of Medicine, Mashhad University of Medical Sciences, Mashhad, IR Iran

**Keywords:** Varicocele, Premature Ejaculation, Varicocelectomy

## Abstract

**Background:**

Premature ejaculation is one of the most problems in clinical practice. The association between varicocele and premature ejaculation was poorly understood. The effects of varicocelectomy on premature ejaculation in varicocele patient was studied.

**Objectives:**

The aim of this study was to determine the impacts of varicocelectomy on patients with both premature ejaculation and varicoceles.

**Patients and Methods:**

This was a clinical trial study, conducted on 124 patients (20-35 years old), with varicoceles and premature ejaculation (PE), since March 2011 to April 2013. Inguinal and sub inguinal varicocelectomy were performed for them. All patients had both impairment of spermiogram and PE. These patients were followed up for about 2 years and evaluated for PE, in addition to parameters of spermiogram, before and after the surgery.

**Results:**

A total number of 124 patients with varicoceles and PE were enrolled into the study. Following the surgery 46 patients (37%) were fully treated (P < 0.001), 78 patients (63%) had improvements in PE symptoms changed to early ejaculation (P < 0.05) and 89 patients (72%) had improved parameters of spermiogram (P < 0.002).

**Conclusions:**

In a significant number of patients who had clinical varicocele and not well responded to medical treatments for PE, varicocelectomy could effectively improve PE and spermiogram parameters.

## 1. Background

Varicocele is one of the most common attributable causes of secondary and a reason for a significant portion of primary infertility in male patients (1). Varicocele is defined by elongated, dilated and tortuous veins draining the testicle and is commonly observed in about 15-20% of the general male population. Varicoceles are generally detected during puberty (1) and the condition is much more common in adolescence and adulthood (2). Varicocelectomy, the surgical varicocele repair surgery and the gold standard treatment of the condition, is a microscopic sub-inguinal procedure. Sub-inguinal refers to the location of the incision (3).

Using the term “microscopic” means that an operating microscope is used by the surgeon, performing the delicate parts of the operation. Another technique of varicocelectomy is the inguinal technique, used for our patients. In this procedure we incise the external oblique fascia and afterwards the spermatic cord will be evaluated for dilated veins by the surgeon, without a microscope. This style is simple and well accepted. Involuntary ejaculation or premature ejaculation (PE) is one of the most prevalent problems in men. Based on a National Health and Social Life Survey report, 21% of American men aged 18 to 59 years are affected by PE. Different reports have shown that less than 5% to more than 30% of men are affected by PE (4, 5). The condition has two major subgroups: primary premature ejaculation (PPE) which happens during the first few years of sexual activity and acquired or secondary premature ejaculation (SPE) which is a lifelong condition (6, 7). However, inflammatory diseases of the prostate, pelvic-nerve damages, malformations of the reproductive system and consumption of some medications like pseudoephedrine can also cause PE (8, 9).

## 2. Objectives

Encountering a significant proportion of patients with PE, also being affected with varicoceles, the authors thought that there may be a relation between PE and varicoceles and to evaluate this relation we decided to conduct a clinical trial research. In this study, conducted on 124 patients with varicoceles and PE, we evaluated the patients regarding their rate of response to varicocelectomy and improvements in spermiograms.

## 3. Patients and Methods

In a clinical trial study conducted in a two year period, from March 2011 to April 2013, we selected 144 patients with clinical varicoceles and PE from the patients referred to the urology clinics of Mashhad Medical Sciences University Hospitals. Left inguinal and sub inguinal varicocelectomy were performed for patients with grade 2-3 varicoceles, impaired spermiogram and PE together. We defined PE as intravaginal ejaculatory latency time less than 1-2 min (IELT < 1-2 min) and early ejaculation as intravaginal ejaculatory latency time between 2-5 min (2 < IELT < 5 min) and fully improvement as intravaginal ejaculatory latency time more than 5 min (IELT > 5 min). These patients had already been on paroxetin and clomipramine for 6 months, for PE treating and had not responded properly. A total number of 20 patients were excluded due to antidepressant drug consumption, having an external genital malformation, urogenital infections, history of cancer or previous history of pelvic surgery. There were 124 patients aged 20-35 years old included in the study, all agreed to participating. We started the study with clinical and paraclinical evaluations (like clinical examination, a sonogram and laboratory tests to find out the grade of the clinical varicoceles and its effects on the spermiogram). PE was also evaluated.

## 4. Results

In this clinical trial study, 124 patients aged 26 ± 4.5 years old, with varicoceles and PE were investigated. Following the varicocelectomy, 46 patients (37%) with PE (intravaginal ejaculatory latency less than 1-2 min) were fully treated with statistically meaningful difference in the ejaculation time (IELT > 5 minutes) (P < 0.001). Another 78 patients (63%) had improved PE changed to early ejaculation (2 < IELT < 5) (P < 0.05) following the varicocelectomy. These two groups were fully satisfied with these improvements. Out of 124 patients undergoing varicocelectomy, 89 patients (72%) had improved spermiogram parameters, following the surgery (P < 0.002).

## 5. Discussion

PE etiology consists of a diverse range of biological and psychological theories. Psychological theories include: the effects of early experience and sexual conditioning, anxiety, sexual technique, the frequency of sexual activity and psychodynamic explanations (10). Based on the possible psychogenic etiologies of PE, the condition is usually treated by clinical psychologists or psychiatrists and the recommended medication often used is one of the serotonin selective reuptake inhibitors (SSRI) and sometimes special neuromuscular exercises like the Kegel’s, which are useful to strengthen the pelvic and sexual muscles (9, 11). Biological explanations for PE include: evolutionary theories, penile hypersensitivity, central neurotransmitter levels and receptor sensitivity (hyposensitivity of the 5-HT2C and/or hypersensitivity of the 5-HT1A receptors suggested for lifelong PE), the degree of arousability, the speed of the ejaculatory reflex and the level of sex hormones (12) and finally lower urinary tract infection like prostatitis. In Murat Gonen et al. report, 51 (77.3%) of the patients with chronic pelvic pain syndrome had PE (13-15), although not enough attention had been paid to the organic genital diseases. In one study by Mr Lotti et al. that investigated the association between varicoceles, premature ejaculation and prostatitis symptoms in Italy, premature ejaculation was the only sexual symptom, significantly associated with varicoceles (29.2% vs. 24.9% in subjects with or without varicocele, respectively: P < 0.05) (16). But in the present study, we evaluated the effects of varicocelectomy and improvements of PE in patients with varicoceles. Observing a lot of patients with varicoceles being also affected by PE, we assumed there might be a relation between these two conditions. To ensure we started this clinical trial research and investigated the relation between varicocelectomy and PE improvement among these patients. It seems that complete clinical evaluation and especially clinical and sonography evaluation of sexual organs are essential for patients with PE, before starting any routine medical treatment. Zohdy et al. studied the effects of varicocelectomy on erectile function and serum total testosterone level, in Egypt. The 5-score international index of erectile function (IIEF) improved significantly in patients with hypogonadism, following the varicocelectomy (17.1 ± 2.6 to 19.7 ± 1.8, P < 0.001), similar to their testosterone levels (379.1 ± 205.8 to 450.1 ± 170.2 ng/dL, P < 0.0001) (17). Similar to the other studies, varicoceles showed to affect the sexual activity of many patients in the present study. It also had significant effects on sexual function of the patients, particularly on PE. In many studies varicocelectomy had improved the sexual satisfaction to different degrees. Our study also showed some degrees of sexual behavior improvement in patients. Altogether, all these facts prove that varicoceles may not only cause infertility, but can also strongly affect the sexual function and quality of life in many patients. It seems that for a significant number of patients with clinical grade 2-3 varicoceles, not well responding to medical treatments for PE (like paroxetin and clomipramine), varicocelectomy can be an alternative which effectively improves PE and spermiogram parameters.
